# Prognostic Nomograms in Oral Squamous Cell Carcinoma: The Negative Impact of Low Neutrophil to Lymphocyte Ratio

**DOI:** 10.3389/fonc.2019.00339

**Published:** 2019-04-30

**Authors:** Davide Mattavelli, Davide Lombardi, Francesco Missale, Stefano Calza, Simonetta Battocchio, Alberto Paderno, Anna Bozzola, Paolo Bossi, William Vermi, Cesare Piazza, Piero Nicolai

**Affiliations:** ^1^Unit of Otorhinolaryngology, Head and Neck Surgery, Department of Surgical Specialties, Radiological Sciences, and Public Health, University of Brescia, Brescia, Italy; ^2^Department of Otorhinolaryngology, Head and Neck Surgery, IRCCS Ospedale Policlinico San Martino, University of Genoa, Genoa, Italy; ^3^Unit of Biostatistics and Biomathematics and Unit of Bioinformatics, Department of Molecular and Translational Medicine, University of Brescia, Brescia, Italy; ^4^Unit of Pathology, Department of Molecular and Translational Medicine, University of Brescia, Brescia, Italy; ^5^Unit of Medical Oncology, Department of Surgical Specialties, Radiological Sciences, and Public Health, University of Brescia, Brescia, Italy; ^6^Unit of Otorhinolaryngology, Maxillofacial, and Thyroid Surgery, Department of Surgery, Fondazione IRCCS, National Cancer Institute of Milan, University of Milan, Milan, Italy

**Keywords:** nomograms, head and neck cancer, oral cancer, squamous cell carcinoma, neutrophil to lymphocyte ratio, prognosis, extranodal extension, perineural spread

## Abstract

**Objectives:** To investigate the prognostic significance of preoperative neutrophil to lymphocyte ratio (NLR) and the impact of different clinical-pathologic factors in a series of primary oral squamous cell carcinomas (OSCC).

**Materials and Methods:** All naive OSCCs treated with upfront surgery between 2000 and 2014 were retrospectively reviewed. Patients with distant metastasis, synchronous head and neck cancer, immunological disorders, or who had received previous chemotherapy and/or radiation of the head and neck area were excluded. The main outcomes were overall (OS), disease-specific (DSS), loco-regional free (LRFS), and distant metastasis free (DMFS) survivals. Univariate (Kaplan-Meier) and multivariate (Cox regression model) analysis were performed, and nomograms developed for each outcome. NLR was analyzed as a continuous variable using restricted cubic spline multivariable Cox regression models.

**Results:** One-hundred-eighty-two patients were included. Five-year estimates for LRFS, DMFS, DSS, and OS were 67, 83.7, 69.5, and 61.2%, respectively. NLR had a complex influence on survival and risk of recurrence: negative for very low and high values, while positive in case of intermediate ratios. At univariate analysis, T classification, 7th AJCC stage, nodal metastasis, perineural spread, and lymphovascular invasion were statistically significant. At multivariate analysis, extranodal extension (ENE) and perineural spread were the most powerful independent prognostic factors. NLR was an independent prognosticator for the risk of recurrence. In nomograms, NLR and ENE had the strongest prognostic effect.

**Conclusions:** In OSCC, very low preoperative NLR values have a negative prognostic impact on survival and recurrence, similarly to high ratios. ENE and perineural spread are the most important clinical-pathologic prognosticators.

## Introduction

Oral squamous cell carcinoma (OSCC) accounts for more than 95% of oral tumors and is the eighth most frequent cancer worldwide, with an estimated incidence of 640,000 new cases per year (1).

Survival of OSCC has slightly improved over the last 30 years, probably as a consequence of multimodal treatment spreading. However, intensified therapeutic regimens can result in significant toxicity and worsen the quality of life of survivors; thus, the definition of reliable prognostic factors is essential to properly stratify the risk of the individual patient and avoid undertreatment as well as unjustified toxicity. For this purpose, a nomogram is an effective way to combine several variables into a single user-friendly tool able to predict outcomes of interest for a given patient.

Recently, a deeper insight on the role of the immune system in the process of cancerogenesis has shed light on the possible prognostic significance of markers of immune activation. Neutrophil to lymphocyte ratio (NLR, i.e., the ratio between the absolute number of circulating neutrophils and lymphocytes) is one of the most promising. An increased count of neutrophils can be considered a marker of tumor-induced systemic inflammation, while lymphocytopenia may reflect a condition of immunosuppression. Therefore, NLR can synthetically render the balance between protumoral inflammatory status and antitumor immune response, where high values of the ratio represent a tumor-induced change of the immune system toward a protumoral pattern.

The negative prognostic impact of high pretreatment NLR values has been investigated for tumors at different sites (2–9). Several studies have shown a similar prognostic value of NLR even for head and neck SCC. Interestingly, the negative impact of higher NLR on both recurrence rate and cancer-related death is comparable to classic clinical-pathologic parameters and is apparently independent of human papilloma virus status (10–16).

The present study is a retrospective analysis of a cohort of patients treated for OSCC in an academic tertiary care center. The aim is to investigate the prognostic significance of preoperative NLR and the impact of different clinical-pathologic factors, and combine the most relevant prognosticators into nomograms to better define the risk profile of an individual patient.

## Materials and Methods

### Patients Cohort

This study was conducted in accordance with the recommendations of the ethics committee of “Spedali Civili” in Brescia named “Comitato Etico Provinciale della Provincia di Brescia.” The protocol (NP-2066-Study WV-H&NCancer) was approved by the abovementioned ethics committee.

We retrospectively reviewed all patients affected by naive OSCC who underwent surgical resection of the primary lesion and synchronous neck dissection (with either an elective or therapeutic intent) between 2000 and 2014 at the Unit of Otorhinolaryngology—Head and Neck Surgery of the University of Brescia, Italy. Exclusion criteria were: (1) presence of distant metastases and/or synchronous head and neck SCC; (2) unavailable follow-up (death within 1 month after completion of treatment or impossibility to have information about the patient status); (3) previous chemotherapy for any cancer and/or radiotherapy in the head and neck area; (4) immunological disorders or immunosuppressed status.

### Blood Samples and Clinical-Pathological Data

Preoperative blood cell counts of all patients were retrospectively retrieved. NLR was defined as the absolute neutrophil count divided by the absolute lymphocyte count. Blood cell counts of a cohort of non-oncologic patients who underwent nasal septoplasty between 2013 and 2014 were included as a control group.

Clinical-pathologic variables analyzed included patient's profile, tumor characteristics, nodal status, and adjuvant treatments. All tumors were classified according to the 7th edition of the TNM staging system.

### Principles of Treatment and Follow-Up

Surgery was always performed upfront with curative intent. Resection was planned according to the site, extension, and tumor depth of invasion (DOI). A purely transoral excision was performed for lesion with DOI < 10 mm; conversely, compartmental surgery with a “pull-through” approach was applied for tongue and oral floor SCC with DOI >10 mm (17). Bone involvement was managed by marginal or segmental mandibulectomy in case of cortical or medullary invasion, respectively.

In case of elective treatment of the neck, the dissection encompassed levels I-III or I-IV, according to the anatomic subsite(s) involved by the tumor. If clinical positive nodes were detected, a comprehensive neck dissection (levels I-V) was planned.

Adjuvant treatment(s) was discussed case by case in the multidisciplinary tumor board. In general, photon beam radiotherapy (60–66 Gy with daily fractions of 1.8–2 Gy) was delivered in case of high-grade tumors, advanced T categories (pT3-T4a), lymphovascular invasion, perineural spread, and/or more than 2 positive nodes. Concurrent chemotherapy (cisplatin 100 mg/mq every 3 weeks, or 40 mg/m^2^ weekly) was administered in case of positive margins and/or extranodal extension (ENE).

Follow-up included clinical examination every 2 months and imaging studies (magnetic resonance [MR] or computed tomography [CT], and neck ultrasonography [US]) every 4–6 months during the first 2 years; clinical and radiological evaluations were subsequently performed every 6 months till the 5th year, and then once yearly. Metastatic spread was ruled out by total body CT or PET-CT every 6 months during the first 2 years, and then yearly.

### Statistical Analysis

We analyzed 4 survival endpoints (overall survival [OS], disease-specific survival [DSS], loco-regional free survival [LRFS], distant metastasis free survival [DMFS]) defined as the time between surgery and the date of the corresponding event (death for any cause, cancer related death, locoregional recurrence, distant metastasis) or last follow-up visit.

Student *t*-test, Chi-square test, Mann-Whitney test and Spearman test were used for group comparisons, as appropriate. Univariate survival curves were estimated using the Kaplan–Meier method and compared by the Log-rank test. Multivariate analyses were performed using Cox proportional hazard models. NLR was modeled as a continuous variable, and its functional relationship with survival outcomes was evaluated by restricted cubic spline using four knots (18). In all analyses a significance level of 5% was used.

To estimate the bivariate distribution of survival times and NLR levels, we computed a nearest-neighbors estimator using a rectangular kernel (19, 20). For illustration purposes, we show the estimated survival curves for low, medium, and high levels of NLR, determined as the survival curve estimate for the neighborhood of the smallest, median, and largest values of the signature, respectively.

The final models were internally validated using non-parametric bootstrap with 200 resamples. The accuracy of predictions was evaluated by estimating the model's calibration and discrimination as measured by the concordance index (c-index). The c-index is the probability that for two randomly selected patients, the patient who experienced the event first has a higher predicted probability of having the event. Therefore, a c-index of 0.5 represents agreement by chance alone, while a c-index of 1 means perfect discrimination. C-index are reported as naive model estimates, their bootstrap estimates (average of individual bootstrap estimates) as well as optimism-corrected estimates.

Calibration curves were drawn by grouping patients with considering their nomogram-predicted probabilities and plotting the mean of predicted probabilities for each group with the mean observed Kaplan–Meier estimate.

SPSS version 23.0, GraphPad Prism and R were used for statistical analysis.

## Results

### Demographics, NLR, and Clinical-Pathologic Variables

One-hundred-eighty-two patients were considered eligible for the study. Median age was 64 years (range, 26–93) with a slight male prevalence (male to female ratio = 1.7).

**Figure 1:**
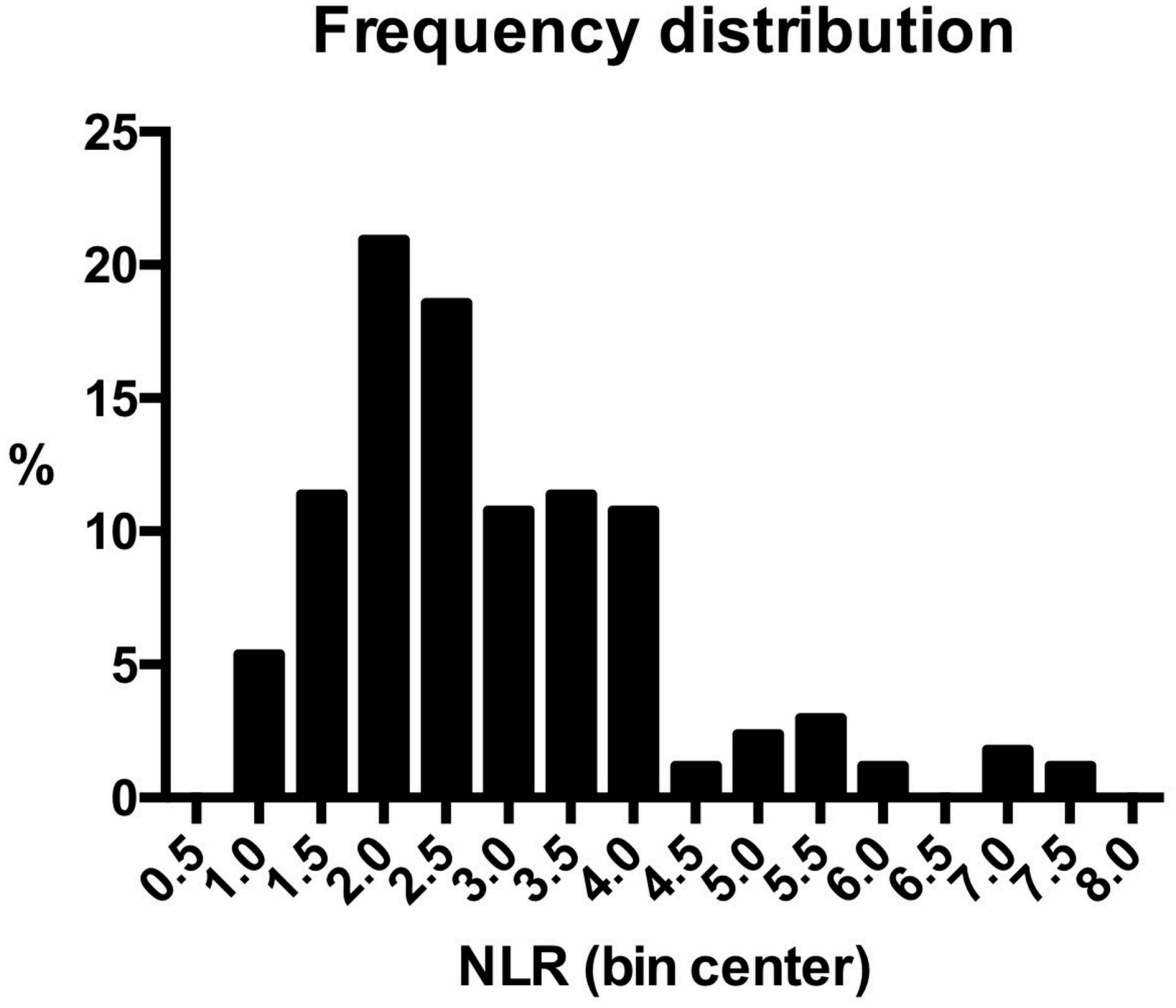
Frequency distribution of neutrophil to lymphocyte ratio values in the series.

NLR was available for 167 (91.8%) patients, because preoperative blood cell count could not be retrieved in the remaining 15. Median value was 2.54 (range, 0.80–7.55). Frequency distribution of NLR values in the series is depicted in Figure 1. The control group was composed of 24 patients who underwent nasal septoplasty. In patients affected by OSCC, neutrophil count was higher (*p* < 0.0001), lymphocyte count lower (*p* = 0.017), and consequently NLR higher (*p* < 0.0001) than in the control group (Table 1).

**Table 1:** Comparison of blood parameters between OSCC patients and control group.

		**Neutrophils**	p^*^	**Lymphocytes**	p^*^	**NLR**	p^*^
	n	10^3^/uL	< 0.0001	10^3^/uL	0.017		< 0.0001
OSCC	167	4.84 ± 1.68		1.82 ± 0.55		2.89 ± 1.33	
Controls	24	3.44 ± 0.66		2.34 ± 0.71		1.62 ± 0.59	

* *Students t-test*.

Clinical-pathologic data are summarized in Table 2. The most frequently involved subsites were the mobile tongue and floor of the mouth, accounting for about 75% of the whole series. At referral, 65.9% of patients presented with advanced disease (7th AJCC stages III-IV) and 42.3% with a high-grade tumor.

**Table 2:** Clinical-pathologic features of the series.

**Variable**		***N***	**%**
Gender	Male	115	63.2
	Female	67	36.8
Behavioral risk factors	None	67	36.8
	Alcohol	11	6
	Smoke	55	30.2
	Smoke and alcohol	43	23.6
Pack/Year	< or = 10	65	35.7
	>10	70	38.5
	Not available	47	25.8
Subsite	Tongue	90	49.5
	Oral floor	46	25.3
	Retromolar trigone	18	9.9
	Alveolar crest	18	9.9
	Cheek mucosa	7	3.8
	Hard palate	2	1.1
	Lips	1	0.5
Treatment	Surgery	78	42.9
	Surgery+RT	61	33.5
	Surgery+RT+CHT	43	23.6
pT	T1	36	19.8
	T2	55	30.2
	T3	6	3.3
	T4	85	46.7
pN	N0	97	53.3
	N1	29	15.9
	N2a	2	1.1
	N2b	42	23.1
	N2c	12	6.6
	N3	0	0
ENE	Absent	43	50.6
	Present	42	49.4
AJCC stage	I	23	12.6
	II	39	21.4
	III	16	8.8
	IV	104	57.1
Surgical margins	Negative	97	53.3
	Close (< 5 mm)	53	29.1
	Positive	32	17.6
Grading	1	18	9.9
	2	87	47.8
	3	77	42.3
Perineural spread	Absent	92	50.5
	Present	90	49.5
Lymphovascular invasion	Absent	131	72
	Present	51	28
Bone invasion	Absent	149	81.9
	Cortical	15	8.2
	Medullary	18	9.9

*CHT, chemotherapy; ENE, extranodal extension; RT, radiotherapy*.

Positive margins were detected in 17.6% of surgical specimens. On average, 47 lymph nodes were removed in each neck dissection. In pN+ patients, the median of positive lymph nodes was 2 (range, 1–18); mean and median nodal ratios were 0.07 and 0.05, respectively (range, 0.01–0.32). Adjuvant treatment(s) was administered in 57.1% of patients.

**Figure 2:**
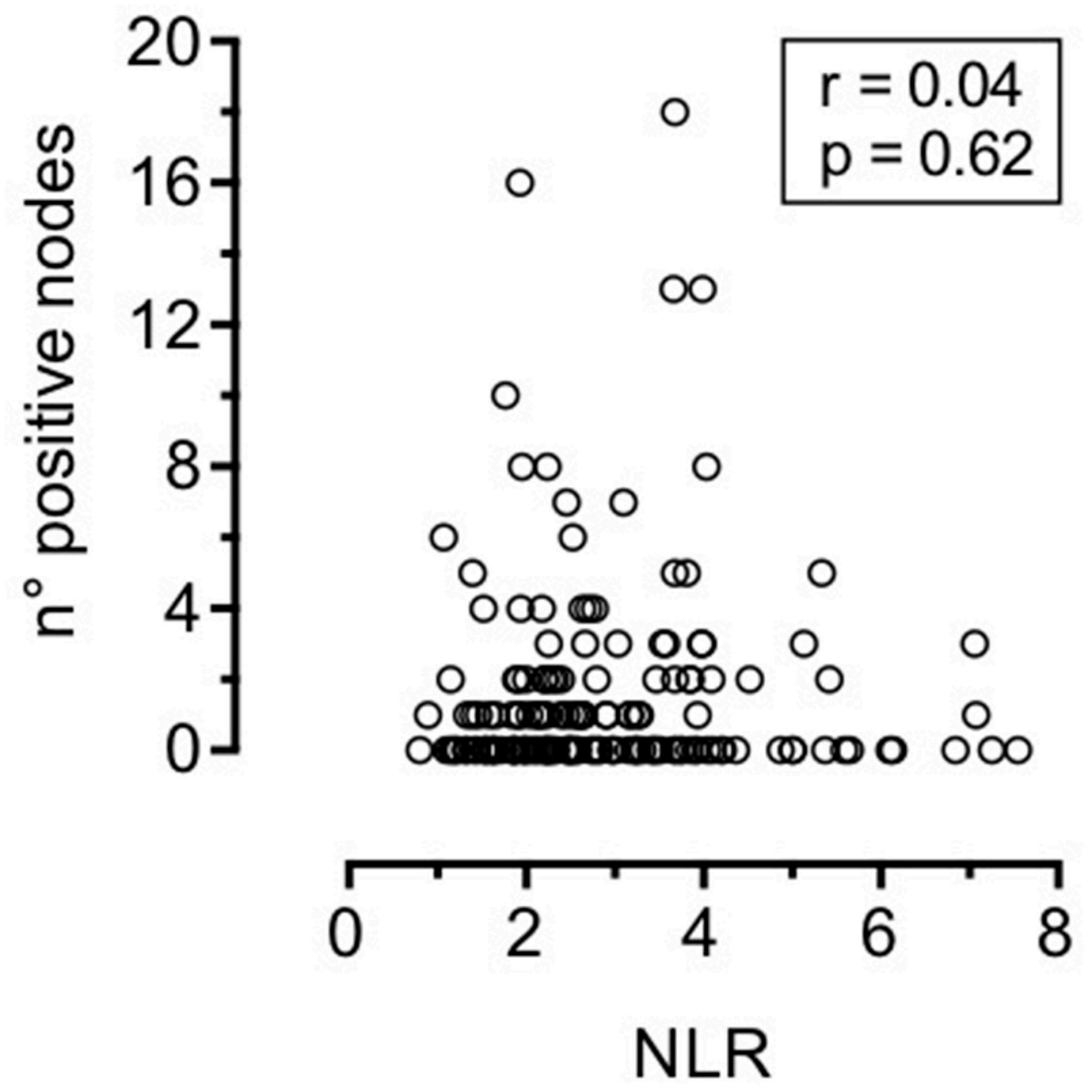
Correlation between neutrophil-to-lymphocyte ratio and number of positive nodes (Spearman test).

Associations between NLR and clinical-pathologic variables are depicted in Table 3 and Figure 2. Patients with advanced T classification tended to show higher NLR values, while no correlations was demonstrated with 7th AJCC stages, number of positive nodes, ENE, perineural spread, and lymphovascular invasion.

**Table 3:** Association analysis between neutrophil-to-lymphocyte ratio and pathological variables; *p*-value estimated by Mann-Whitney test.

**Variables**		***N* (%)**	**NLR**	
			**median (I-III Q)**	***p***
pT	T1-2	86 (51)	2.40 (1.85–3.28)	**0.036**
	T3-4	81 (49)	2.70 (2.22–3.68)	
7^th^ AJCC stages	I-II	58 (35)	2.32 (1.71–3.31)	0.077
	III-IV	109 (65)	2.65 (2.12–3.67)	
ENE (N^+^)	Absent	40 (52)	2.53 (1.95–3.21)	0.298
	Present	37 (48)	2.63 (2.18–3.68)	
PNI	Absent	83 (50)	2.51 (1.88–3.40)	0.326
	Present	84 (50)	2.62 (2.03–3.75)	
LVI	Absent	122 (73)	2.50 (1.93–3.44)	0.279
	Present	45 (27)	2.66 (2.10–3.84)	

*Bold indicates p values inferior to 0.05 (statistical significant)*.

### Oncological Outcomes

Mean follow-up was 54 months (range, 2–163). Overall, recurrence rate was 39.6%: 42 (23%) local, 37 (20.3%) regional, and 29 (15.9%) distant relapses were recorded. Thirty-two (17.6%) patients had multiple recurrences. Seventy-eight (42.9%) patients died during follow-up; 53 (29.1%) deaths were cancer-related.

Two and 5-year estimates for LRFS, DMFS, DSS, and OS were 71.7 and 67%, 87.5 and 83.7%, 81.5 and 69.5%, 76.9 and 61.2%, respectively.

**Figure 3:**
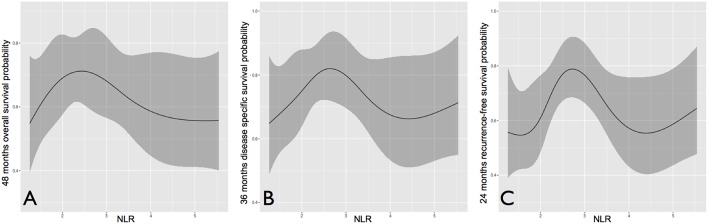
Overall **(A)**, disease-specific **(B)**, and recurrence-free survival **(C)** probability according to neutrophil to lymphocyte ratio, analyzed as a continuous variable.

NLR was first studied as a continuous variable in univariate analysis to investigate its global influence on survival probabilities, and showed a bimodal prognostic impact. In each outcome analyzed, it tended to form a bell-shaped curve with an asymmetric peak. In fact, an increased risk for death and/or recurrence was associated with very low and high values of NLR, while patients with central NLR values (approximately in between 2 and 4) tended to show better oncologic outcomes (Figure 3).

**Figure 4:**
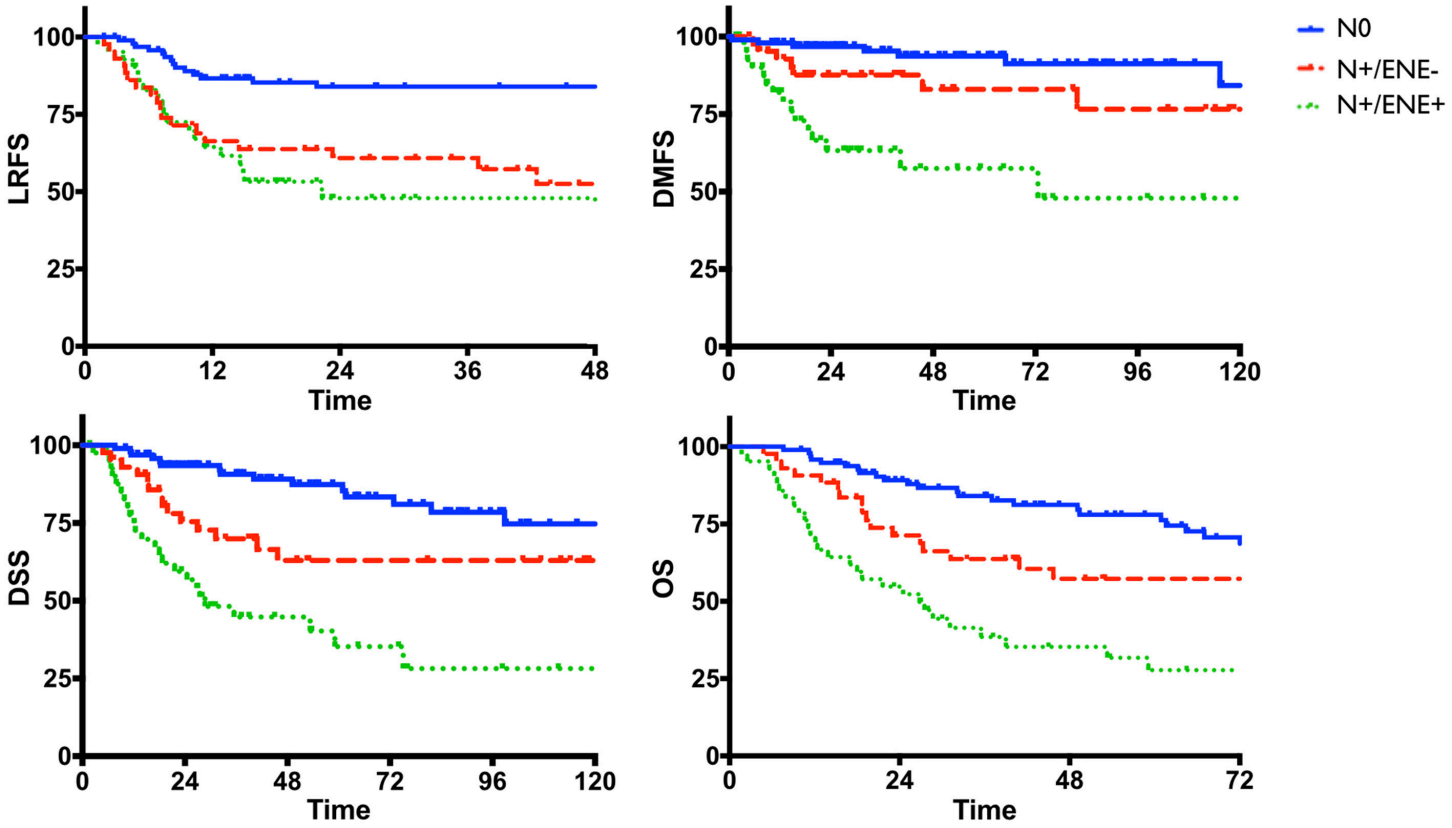
Survival estimates (Kaplan-Meier) according to nodal status.

Univariate analysis of clinical-pathologic prognosticators is summarized in Table 4. T classification, 7th AJCC stage, nodal status, perineural spread, and lymphovascular invasion were the strongest prognosticators. In particular, the presence of ENE resulted in a remarkable further decrease of survival estimates in patients with nodal metastasis (Figure 4).

**Table 4:** Univariate analysis.

**Variable**		**5 y LRFS**		**5y DMFS**		**5y DSS**		**5y OS**	
		**%**	***p***	**%**	***p***	**%**	***p***	**%**	***p***
Age	< 65	61.3	0.598	82.2	0.348	73.7	0.166	67.5	**0.030**
	≥ 65	68.7		86.0		63.8		50.9	
Gender	Male	71.4	**0.025**	83.1	0.909	69.4	0.897	58.7	0.465
	Female	36		84.4		69.3		65.4	
Subsite	Tongue- oral floor	66.6	0.656	84.6	0.374	73.4	0.266	63.5	0.300
	TRM- Alveolar crest	54.2		76.2		53		47.4	
	Other	67.5		100		85.7		85.7	
pT	T1-T2	74.1	**0.006**	92.1	**0.002**	79	**0.004**	70.6	**0.003**
	T3-T4	37		74.5		59		50.6	
pN/ENE	N0	82.4	** < 0.0001**	93.7	** < 0.0001**	77.4	** < 0.0001**	78	** < 0.0001**
	N+/ENE-	26.3		83		63		57.3	
	N+/ENE+	0		57.5		35.2		27.8	
Stage	I-II	88.4	** < 0.0001**	95.9	**0.001**	92.2	** < 0.0001**	83.5	**0.0001**
	III-IV	51		76.8		57.4		49.3	
Surgical margins	Negative	66.6	0.455	86.3	**0.054**	72.4	0.192	65.3	0.063
	Positive	53.4		70.7		53.5		41.8	
Grading	G1	87.2	**0.030**	88.9	0.335	80.9	0.308	67.5	0.266
	G2	66.9		77.8		68		58.5	
	G3	37.5		88.7		66.4		61.8	
Perineural spread	Absent	73.4	**0.013**	92	**0.0006**	80.1	**0.004**	68.6	0.079
	Present	43.8		74.6		58		53.3	
Lymphovascular invasion	Absent	64.4	0.216	89.7	**0.001**	75.6	**0.0008**	68.6	**0.001**
	Present	66.9		66.1		48.3		38.2	
Bone invasion	Absent	71.1	**0.003**	84.1	0.465	73.2	**0.057**	63.5	0.161
	Cortical	62.9		92.9		78.8		65.7	
	Medullary	0		72.6		39.1		39.1	
^*^Nodal ratio	< 0.07	56.3	0.206	70.24	** < 0.0001**	58.1	**0.023**	49.4	0.066
	≥ 0.07	39.5		0		29.3		26.9	
	< 0.1	56.2	**0.040**	67.7	** < 0.0001**	57.7	**0.002**	49.5	**0.009**
	≥ 0.1	31.2		0		12.8		11.9	
^*^Number of positive nodes	1-2	52.4	0.223	#86.4	**0.007**	61.8	**0.001**	55.8	**0.001**
	3-4	53.4		#63.9		42.1		33.7	
	≥ 5	38.8		#59.3		13.7		11.8	

ENE, extranodal extension; N+, node metastasis; RMT, retromolar trigone;

* *Analysis performed in the subgroup of patients with nodal metastasis (n = 85); # Survival at 2 years*.

*Bold indicates p values inferior to 0.05 (statistical significant)*.

**Figure 5:**
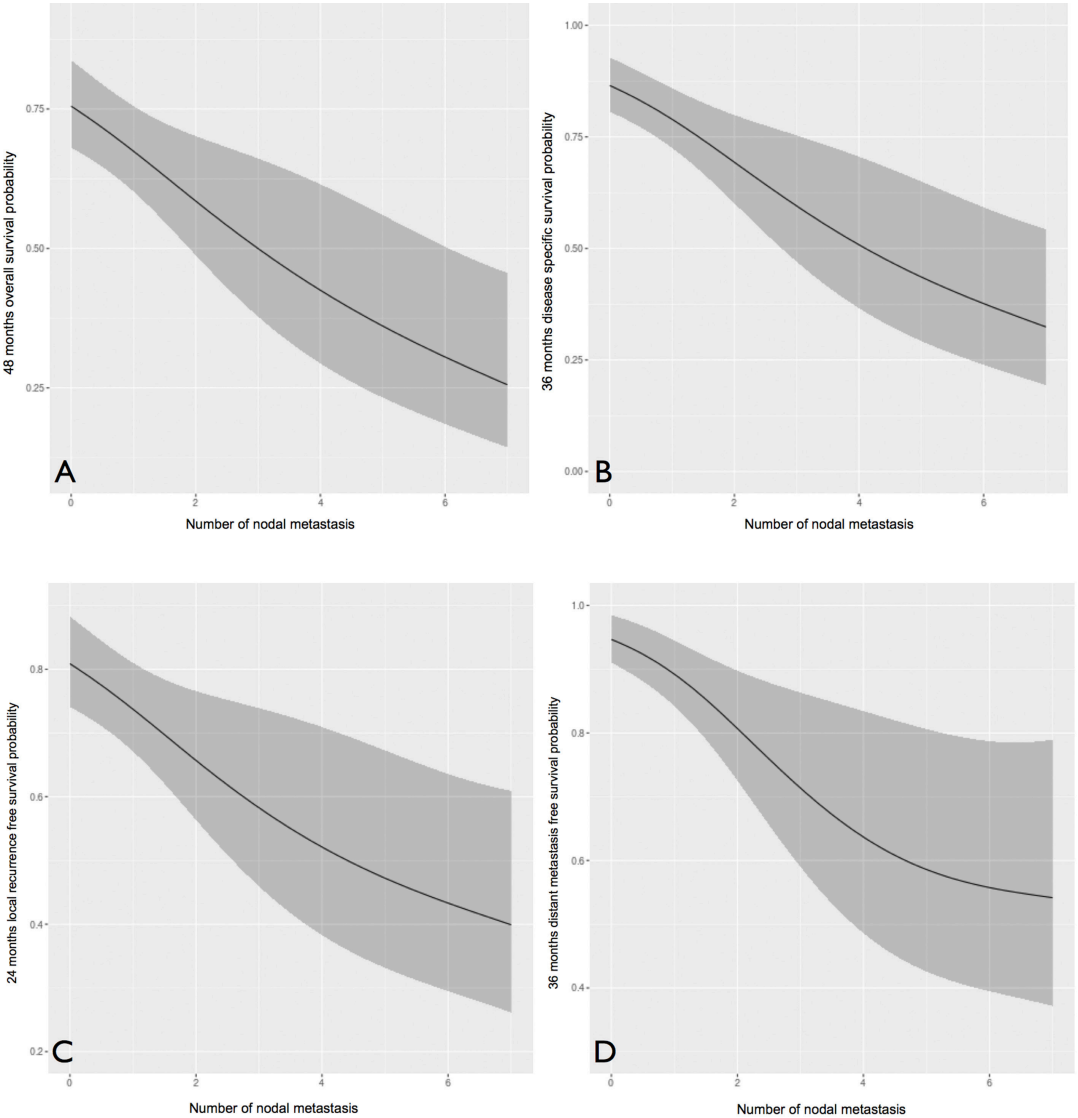
The influence of number of positive nodes (analyzed as a continuous variable) on overall **(A)**, disease-specific **(B)**, local recurrence-free **(C)**, and distant metastasis-free **(D)** survival probability. In all cases, an inverse proportion relationship is evident.

**Figure 6:**
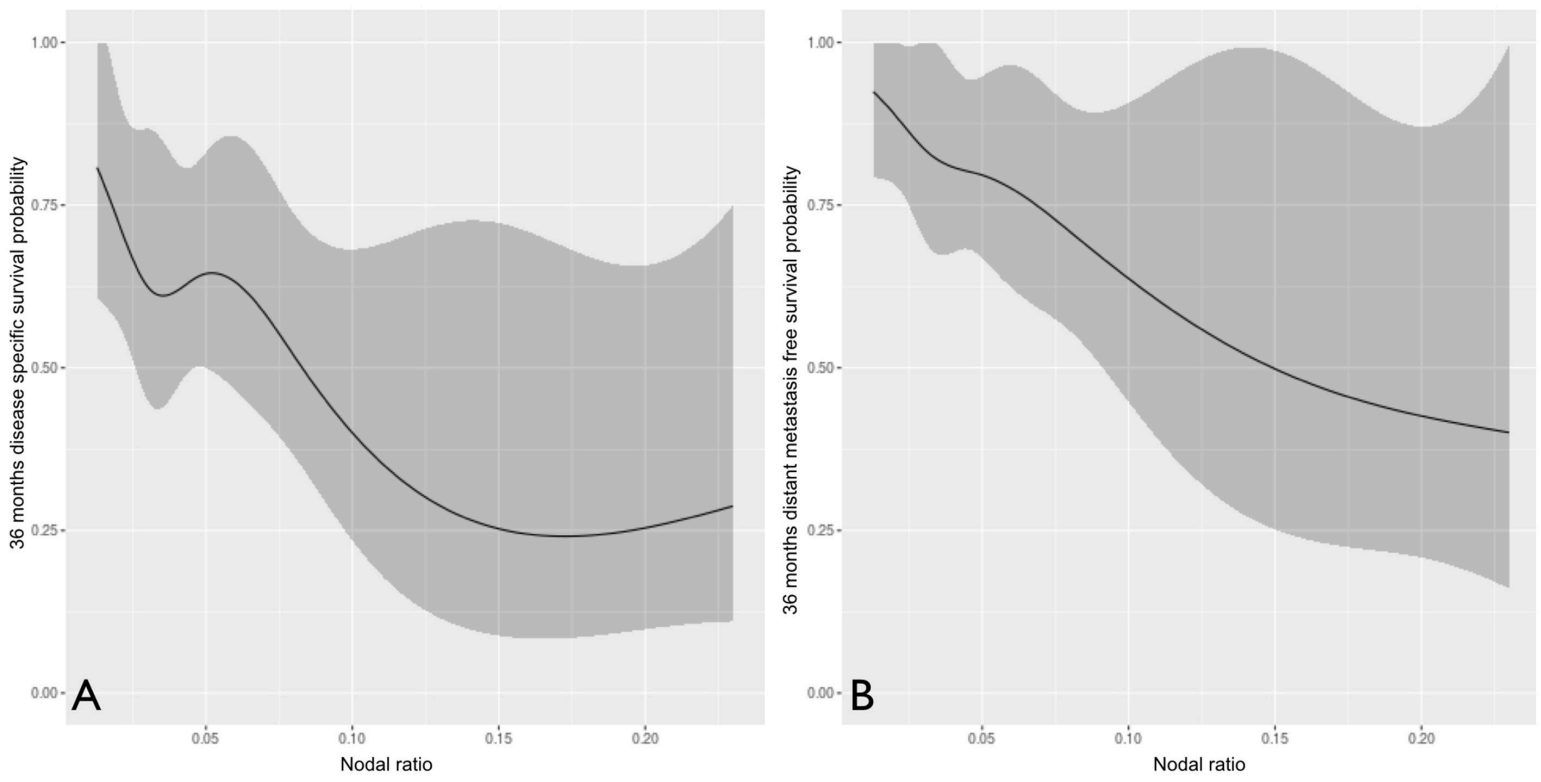
The influence of nodal ratio (analyzed as continuous variable) on disease-specific **(A)** and distant metastasis-free **(B)** survival probability.

We further analyzed the number of positive nodes (Figure 5) and nodal ratio (Figure 6) as continuous variables. The curves demonstrate an inverse relationship between these variables and each outcome analyzed. In fact, the increase in the number of positive nodes or in the value of nodal ratio resulted in a proportionally decreased probability of disease control and survival.

In multivariate analysis (Table 5), nodal status was the most powerful independent prognosticator, and the remarkable worsening of disease control and survival conferred by ENE was confirmed. Perineural spread was an independent risk factor for distant metastasis (HR 4.39) and cancer-related death (HR 2.02), but not for local recurrence. The prognostic impact of NLR on recurrence, as depicted in Figure 3, was independent of other major clinical-pathological variables (LRFS, *p* = 0.005; DMFS, *p* = 0.046), while it was close to significance for DSS (*p* = 0.078).

**Table 5:** Multivariate analysis.

		**LRFS**		**DMFS**		**DSS**		**OS**	
		**HR (95% CI)**	***p***	**HR (95% CI)**	***p***	**HR (95% CI)**	***p***	**HR (95% CI)**	***p***
pT (T3-T4 vs. T1-T2)		1.56 (0.83–2.92)	0.169	2.64 (1.06–6.56)	**0.037**	1.6 (0.83–3.07)	0.162	1.53 (0.89–2.62)	0.126
pN/ENE	pN0	1		1		1		1	
	pN+ ENE-	3.95 (1.92–8.12)	** < 0.001**	1.77 (0.6–5.23)	0.302	2.64 (1.19–5.83)	**0.017**	1.76 (0.94–3.3)	0.080
	ENE+	4.08 (1.96–8.48)	** < 0.001**	6.82 (2.62–17.75)	** < 0.001**	5.97 (2.84–12.54)	** < 0.001**	3.83 (2.12–6.94)	** < 0.001**
Perineural invasion		1.26 (0.71–2.24)	0.426	4.39 (1.76–10.95)	**0.002**	2.02 (1.09–3.73)	**0.025**	1.47 (0.89–2.41)	0.129
NLR^*^		–	**0.005**	–	**0.046**	–	0.078	–	0.176

*ENE, extranodal extension; HR, hazard ratio; CI, confidence interval; N+, node metastasis*.

* *Neutrophil to lymphocyte ratio (NLR) was modeled using a restricted cubic spline with 3 knots placed at 10, 50, and 90% quantiles. Bold indicates p values inferior to 0.05 (statistical significant)*.

**Figure 7:**
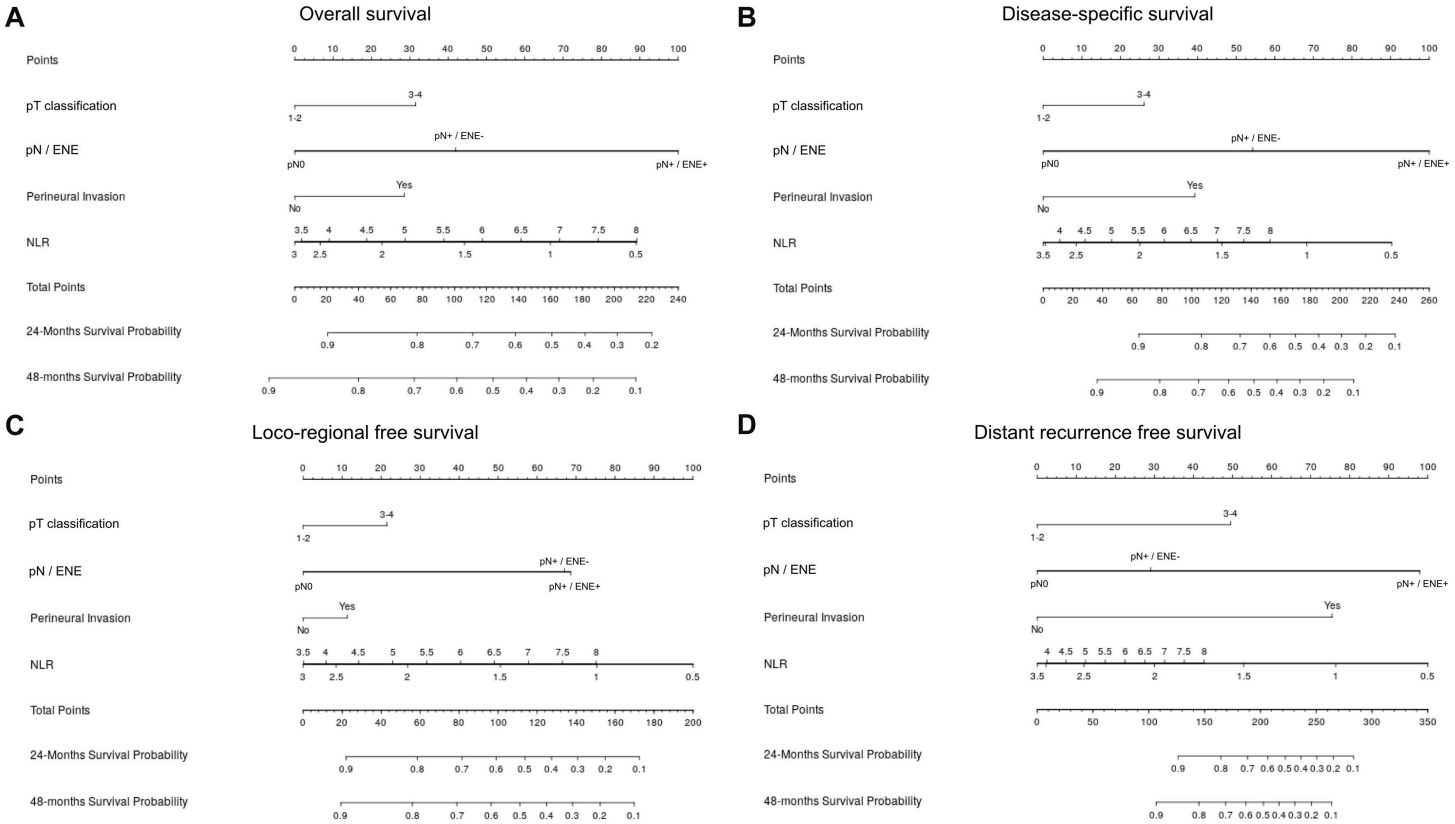
Nomogram for prediction of overall survival **(A)**, disease-specific survival **(B)**, loco-regional free survival **(C)**, and distant metastasis-free survival **(D)**. In a nomogram, a score on a scale from 1 to 100 is assigned to each covariate based on its predictive contribution to the model. Briefly, the variable that displays the strongest prognostic effect is given 100 points, and all the others receive a smaller value proportional to their size effect. The total amount of points of the single case is correlated to survival probability of the individual patient (axis in the bottom of each figure).

Lastly, we developed prognostic nomograms for OS (Figure 7A), DSS (Figure 7B), LRFS (Figure 7C), and DMFS (Figure 7D) from the results of Cox proportional hazards regression analysis.

**Figure 8:**
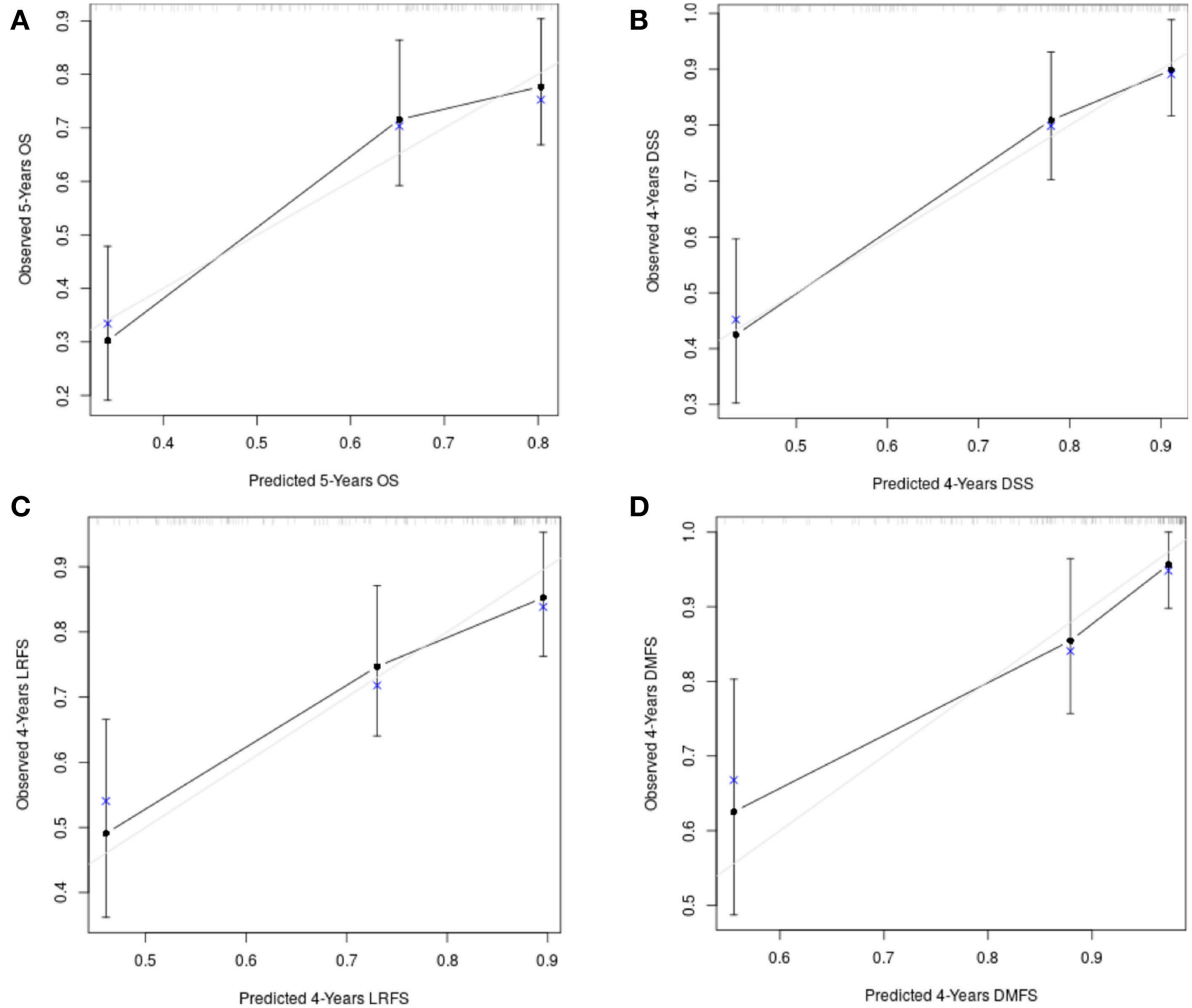
The calibration plots for the survival models: **(A)** overall survival; **(B)** disease-specific survival; **(C)** loco-regional free survival; **(D)** distant metastasis free survival.

Figure 8 shows the calibration plots for the survival models (OS, DSS, LRFS, and DMFS), in which the predicted probability of survival is plotted against the observed data.

Model discrimination was evaluated with the c-index, which quantifies the level of concordance between the predicted and observed survivals. The c-index for the final models and their bootstrap estimates and confidence intervals were 0.71 (95% CI, 0.66 to 0.77), 0.76 (95% CI, 0.68 to 0.81), 0.73 (95% CI, 0.65 to 0.80), 0.81 (95% CI, 0.73 to 0.88) for OS, DSS, LRFS, and DMFS, respectively. The optimism corrected c-indices generated with the boostrap validation were 0.68 (95% CI, 0.62 to 0.73), 0.73 (95% CI, 0.67 to 0.80), 0.68 (95% CI, 0.62 to 0.75), 0.76 (95% CI, 0.68 to 0.84) for OS, DSS, LRFS, and DMFS, respectively.

### Subgroup Analysis

**Figure 9:**
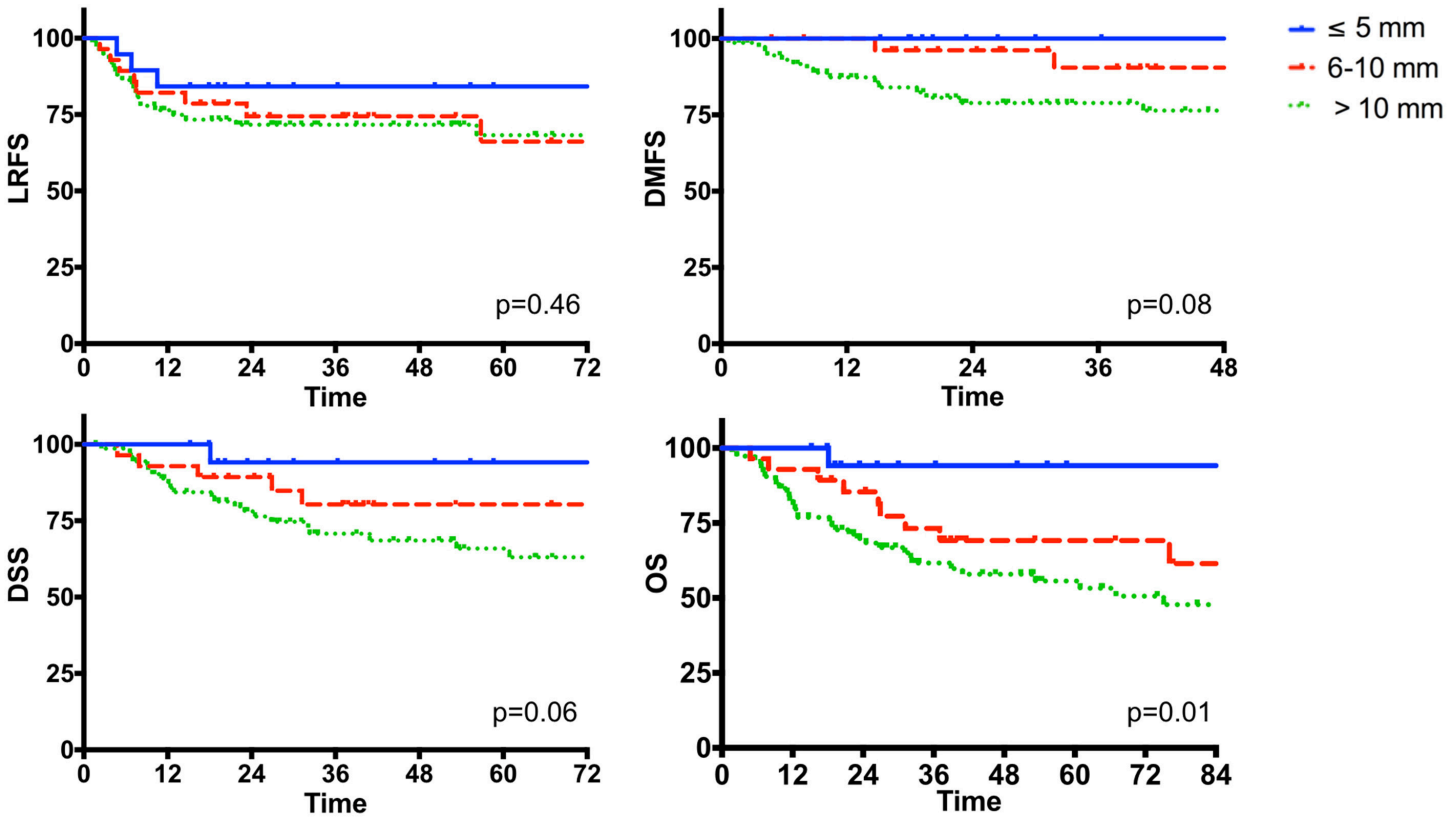
The impact of the depth of invasion in the subgroup of oral cancer originating from the mobile tongue and oral floor (*n* = 136).

DOI was analyzed in the subgroup of tumors originating from the mobile tongue and oral floor, and was available in 121 out of 136 (88.9%) patients. Mean and median DOI were 13.9 and 12.5 mm, respectively (range, 1–38). Stratification into 3 categories [ ≤ 5, 5–10, and >10 mm as recently suggested by the 8th Edition of the AJCC UICC TNM Staging System (21) for T1, T2, and T3 OSCC] identified three different classes of risk for distant relapse and death (Figure 9).

Lastly, we explored the impact of adjuvant treatment(s) in patients with ENE (*n* = 42). In univariate analysis, we compared patients undergoing concurrent chemoradiotherapy (*n* = 22) with those who received radiotherapy alone (*n* = 15). Five patients were excluded because they did not receive any adjuvant treatment(s). The two groups were balanced regarding age, pT and pN categories, margin status, grading, perineural and lymphovascular invasion (*p* = n.s.), while patients undergoing concurrent chemoradiotherapy were significantly younger (*p* = 0.003; Supplementary Table 1).

**Figure 10:**
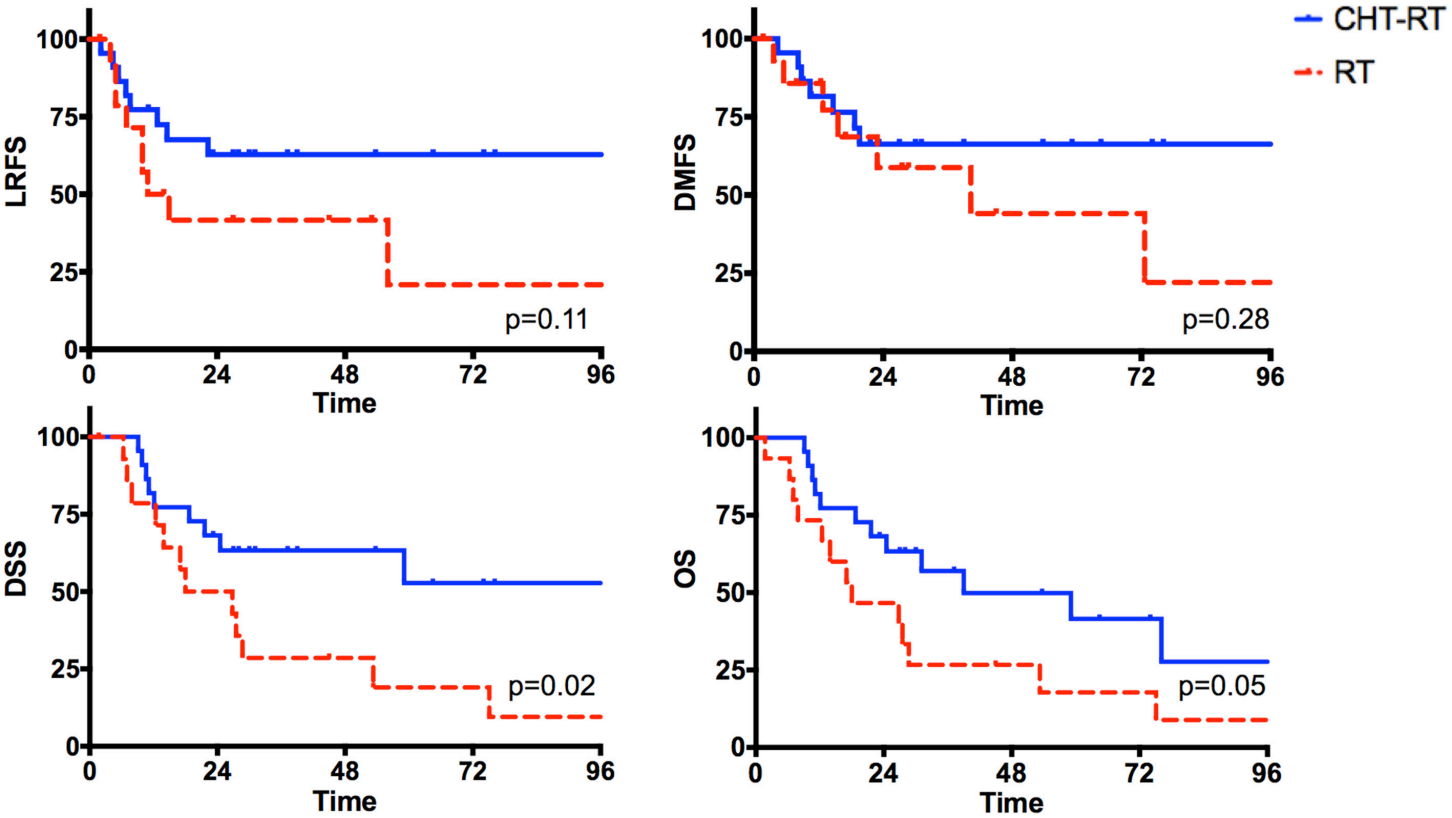
The impact of adjuvant treatments (radiotherapy alone vs. concurrent chemoradiotherapy) in the subgroup of patients with extranodal extension.

Chemotherapy was associated with a remarkable improvement of disease control and survival (Figure 10). In fact, patients who received radiotherapy alone experienced almost a 3-fold higher risk of cancer-related death (HR 2.78; CI, 1.13-6.84; *p* = 0.02) resulting in a remarkable decrease of 5-year DSS (19 vs. 52.8%). A similar trend was found for OS (*p* = 0.05).

## Discussion

The most relevant findings of our study are: (1) NLR displays a complex influence on survival and risk of recurrence, which is mostly independent of other clinical-pathological variables: worst outcomes are associated to low and high values, while better estimates are found with intermediate ratios; (2) ENE, number of positive nodes, and perineural spread are the most powerful clinical-pathologic prognosticators; (3) NLR and ENE display the strongest prognostic effects in nomograms.

The main strength of our series is its homogeneity in terms of patient and tumor profiles as well as treatment strategy. All patients indeed received the same surgical procedure (resection of the tumor and neck dissection) as primary treatment at referral in a single center, and adjuvant treatment was planned according to quite homogeneous indications. Likewise, possible modifiers of tumor biological aggressiveness (recurrent disease, synchronous tumors, previous radiotherapy in head and neck, immunosuppression) were excluded. Therefore, selection biases of retrospective studies were minimized.

Survival estimates compare well with those reported in the literature (22–25). Recurrences mostly occurred loco-regionally (31.3%), even though the percentage of distant metastases was not negligible (15.9%).

NLR has several advantages: it is easily and objectively measured, cost effective, and, most importantly, always available before treatment planning.

In accordance with other reports (26, 27), patients affected by OSCC presented with a significantly different white cells count than healthy controls: higher neutrophils, lower lymphocytes, and, consequently, higher NLR. In a recent report, NLR was found to be significantly higher in OSCC even compared to non-malignant oral cavity lesions, suggesting a new tool that might help in identifying oral cavity lesions at higher risk for harboring malignancy (28).

In our study, we analyzed NLR as a continuous variable using restricted cubic spline with four knots in order to avoid any loss of information about its influence on survival. So far, it has been mostly dichotomized using the receiver operating characteristic curve or the median, or even subdivided into tertiles; in a single, recent paper NLR was studied as a continuous variable after logarithmic transformation (29). In recent meta-analyses on the prognostic role of NLR in OSCC, its general interpretation as a bimodal prognostic factor was confirmed (the higher the value, the poorer the survival); cut-offs ranged from 1.77 to 5, but most were around 2.5 (30–32).

Some data in our analysis are new and surprising. In particular, in each outcome the negative prognostic influence of very low values of NLR parallels the well-known impact of high NLR (Figure 3). In nomograms, the prognostic effect of low NLR is even greater than high NLR, and is comparable (and sometimes superior) to ENE (Figure 7). More importantly, it is independent of other clinical-pathological factors with regards to the risk of recurrence, suggesting that NLR might be considered as an adjunctive parameter in the decision-making process for adjuvant treatments.

This finding is unreported in the head and neck cancer literature. However, in a recent publication on 1,335 gastric cancers, Urabe et al. (33) demonstrated a non-linear association between preoperative NLR and survival outcomes by subdividing the series into quartiles: Q1 (NLR < 1.59) showed poorer estimates than Q2 (1.59 < NLR < 2.11) and Q3 (2.11 < NLR < 2.96), but better than Q4 (NLR >2.96), depicting an influence of NLR on survival that is very similar to the one found herein.

The interpretation of this influence of NLR on survival is challenging, and currently only some speculations can be offered. First, it is not biased by an unbalanced distribution of NLR values in the series. In fact, median value of NLR is 2.54, and the group of patients with NLR inferior to 2 is well represented (about 27%; Figure 1). In a recent retrospective analysis on 1202 OSCC, Chen et al. (34) found a double cut-off of NLR (1.94 and 3.66). They then divided the population into three groups at low (< 1.94), intermediate (1.94–3.66), and high (>3.66) risk, which could be considered a sort of surrogate of our approach to NLR as a continuous variable. Their findings are in contrast with ours, as they did not recognize any negative influence of low NLR. However, some differences in patient characteristics need to be mentioned. In the Chinese study, a higher prevalence of low stage, N0 patients is evident, and in this subgroup NLR is significantly lower. Conversely, in our series about 60% of patients with NLR inferior to the median presented with an advanced stage tumor. Therefore, a low NLR value might have a different implication according to tumor stage, and possibly identify two different steps of tumor-host interaction. In early stage cancers, a low NLR value may indicate a limited influence of neoplastic cells on the immune system, similar to healthy controls who showed a comparable low NLR value. In such a subset of patients, prognosis is excellent. Conversely, in high stage tumors very low NLR may be marker of an advanced phase of immune escape, where the immune system is exhausted by a prolonged corruption by cancer cells. The different length of live of neutrophils and lymphocytes may justify from a mathematic perspective the steady decrease of the ratio: neutrophils count reduces in a shorter time, while circulating lymphocytes persist, but with a reduced (or null) antitumoral activity. In this view, “very low” NLR may be the step forward to “high” NLR, as also suggested by our nomograms, where the negative prognostic effect of the former is higher than in the latter. This hypothesis is supported by the work of Wu et al., who analyzed a series of 262 cT1-T2N0 OSCCs and found that high NLR (>2.95) significantly correlated with occult node metastasis, perineural invasion, and tumor thickness >5 mm, while low NLR was associated with better disease-free, disease-specific, and overall survival estimates (35).

Finally, it is noteworthy that the absolute count of circulating neutrophils and lymphocytes is a rough data, and no details about the subpopulations and activation state of leukocytes are available. For example, low activation of neutrophils and an increase in CD4/CD8 ratio have been associated with poorer survival in OSCCs (26). Likewise, a prevalent activation of CD4+ Treg lymphocytes can decrease immunosurveillance and promote a protumoral switch of the immune system in cancer patients (36). Lastly, it would be interesting to investigate the immunologic infiltrate in the primary lesion of these patients. Caldeira et al. demonstrated a differential infiltration of neutrophils and lymphocytes in T1-T2 vs. T3-T4 OSCCs, with a higher density of neutrophils in the intratumoral region and higher neutrophil/lymphocyte ratios in the invasive front of advanced lesions (37). A very low NLR allegedly correlates with an unfavorable pattern of tumor infiltrating lymphocytes and/or tumor infiltrating myeloid cells. Further investigations are warranted to confirm this trend and clarify the reasons that might explain it.

Concerning clinical-pathologic factors, the most important prognosticators were ENE, number of positive nodes, and perineural spread. The negative impact of ENE has been recently fully incorporated in the new TNM staging system (21), where it leads to upstaging of the N category, whatever the size, number, or laterality of positive node(s). Instead, the number of positive nodes is probably an underestimated parameter in the current TNM staging system, since in our analysis it was indeed extremely effective in describing a proportional decrease of survival estimates (Figure 5). The well-established indication to intensify treatment (adjuvant chemoradiation) in case of ENE is confirmed in our series. In fact, the lack of chemotherapy administration (because of comorbidities, or because they were treated in the early study period) had a detrimental effect on every survival end-point. Even though the retrospective nature of the study and the low number of patients in these two subgroups compel one to be cautious, the remarkable difference in outcomes confirm the current trend of a wise but more liberal use of chemotherapy in association with radiotherapy in comorbid patients, and prompt to develop new strategies to tackle tumor with more ominous prognosis, such as the integration of immunotherapeutic strategies into the treatment plan.

Overall, the description of survival probability in the single patient undoubtedly requires assessment of many different prognosticators. In this view, the nomogram can be an effective, quick, and practical tool to favor personalization of treatment and improve patient counseling.

In our model, NLR showed a prognostic effect comparable to other major pathological prognosticators. In accordance with our findings, other published evidence supports NLR as an essential complement in the thorough definition of tumor risk profile. Recently, Lee et al. proposed a new prognostic scoring system for OSCC incorporating the same variables used herein, which was more effective than the AJCC TNM based model (38). Likewise, Kao et al. developed a nomogram for OS in OSCC, in which a new score combining albumin and NLR was shown to increase the prognostic prediction of the model. Interestingly, in their cohort a large prevalence of betel-induced OSCC was present, suggesting that NLR may be an effective prognosticator independently from the specific carcinogenetic process considered (39).

Some limitations of our study should be mentioned. Although our series had strict inclusion criteria, its limited size may weaken our observations. If compared to other nomograms for OSCC proposed in literature (40), our model has the limitation of including pathological prognosticators, which hinders its application at referral and suggest its use mostly in the decision-making for adjuvant treatments. However, this issue is minimized by the great accuracy provided by current radiological studies, which can guarantee an excellent correspondence between clinical and pathological TNM classification, and the common indication for surgery upfront in the treatment of OSCC.

Even though internal validation was performed to avoid over-interpretation of data, external validation in a larger and different cohort is warranted to confirm the reliability and applicability of our results.

Future research should investigate new prognosticators in the field of tumor-host interaction, such as the quality and localization of the immune infiltrate, with the aim to improve definition of the risk profile of the single patient, and customize treatment accordingly.

## Conclusions

In OSCC, very low preoperative NLR showed a negative prognostic impact comparable to its high value. The negative influence of NLR on the risk of recurrence is independent of other major clinical-pathologic prognosticators. In nomograms, NLR and ENE displayed the greatest prognostic effect.

## Author Contributions

DM, DL, FM, WV, CP, and PN designed the study. DM, FM, AP, AB, and SB were involved in data acquisition. SC, AP, FM and DM performed the statistical analysis. DM edited the manuscript, and then DL, FM, WV, PB, CP, and PN corrected and reviewed it. All authors discussed and interpreted the results and gave their final approval to the definitive manuscript.

### Conflict of Interest Statement

The authors declare that the research was conducted in the absence of any commercial or financial relationships that could be construed as a potential conflict of interest.
