# Supplementary material for: Prognostic Nomograms in Oral Squamous Cell Carcinoma: The Negative Impact of Low Neutrophil to Lymphocyte Ratio
Source: Front Oncol. 2019 Apr 30;9:339. doi: 10.3389/fonc.2019.00339 (PMC6503119; doi:10.3389/fonc.2019.00339)
Supplement: Supplementary file 1 [file Table_1.DOCX]

**Supplementary Tab. 1.** Subgroup analysis of patients with extranodal extension. Comparison between the groups undergoing only adjuvant radiotherapy (RT, n=15) or concurrent chemoradiotherapy (CHT-RT, n=22) according to the main clinical-pathologic variables of the series. P values were estimated by ^a^Fisher’s exact, ^b^Chi square, and ^c^Mann-Whitney tests.

|  | | **RT** | **RT-CHT** | **p** |
| --- | --- | --- | --- | --- |
|  |  | N (%) | N (%) |  |
| **Gender** |  |  |  | 0,20^a^ |
|  | Male | 14 (93) | 16 (73) |  |
|  | Female | 1 (7) | 6 (27) |  |
| **Age** |  |  |  | 0,003^c^ |
|  | median (I-IIIQ) | 74.0 (59.5-79.5) | 61.0 (50.0-65.0) |  |
| **pT stage** |  |  |  | 0,73^b^ |
|  | T1 | 1 (7) | 2 (9) |  |
|  | T2 | 1 (7) | 3 (14) |  |
|  | T3 | 2 (13) | 1 (5) |  |
|  | T4 | 11 (73) | 16 (73) |  |
| **pN stage** |  |  |  | 0,55^b^ |
|  | N1 | 1 (7) | 2 (9) |  |
|  | N2b | 12 (80) | 14 (64) |  |
|  | N2c | 2 (13) | 6 (27) |  |
| **Margins** |  |  |  | 0,63^b^ |
|  | Negative | 8 (53) | 9 (41) |  |
|  | Close | 2 (13) | 7 (32) |  |
|  | Positive | 5 (33) | 6 (27) | 0,75^a^ |
| **Grading** |  |  |  |  |
|  | G2 | 7 (47) | 9 (41) |  |
|  | G3 | 8 (53) | 13 (59) |  |
| **Perineural Invasion** |  |  |  | 0,19^a^ |
|  | yes | 6 (40) | 14 (64) |  |
|  | no | 9 (60) | 8 (36) |  |
| **Vasculal Invasion** |  |  |  | 1,00^a^ |
|  | yes | 8 (53) | 12 (55) |  |
|  | no | 7 (47) | 10 (45) |  |
